# An Optimized PZT-FBG Voltage/Temperature Sensor

**DOI:** 10.3390/mi16020235

**Published:** 2025-02-19

**Authors:** Shangpeng Sun, Feiyue Ma, Yanxiao He, Bo Niu, Cheng Wang, Longcheng Dai, Zhongyang Zhao

**Affiliations:** 1 Electric Power Research Institute, State Grid Ningxia Power Co., Ltd., Yinchuan 750011, China; shangpengsun@163.com (S.S.); fyma.1986@163.com (F.M.); svc_hvdc@163.com (B.N.); 18073619762@163.com (L.D.); 2 Liangjiang International College, Chongqing University of Technology, Chongqing 401135, China

**Keywords:** FBG, PZT, voltage, temperature

## Abstract

The piezoelectric grating voltage sensor has garnered significant attention in the realm of intelligent sensing, attributed to its compact size, cost-effectiveness, robust electromagnetic interference (EMI) immunity, and high network integration capabilities. In this paper, we propose a PZT-FBG (piezoelectric ceramic–fiber Bragg grating) voltage–temperature demodulation optical path architecture. This scheme effectively utilizes the originally unused temperature compensation reference grating, repurposing it as a temperature measurement grating. By employing FBGs with identical or similar parameters, we experimentally validate two distinct optical path connection schemes, before and after optimization. The experimental results reveal that, when the input voltage ranges from 250 V to 1800 V at a frequency of 50 Hz, the goodness of fit for the three fundamental waveforms is 0.996, 0.999, and 0.992, respectively. Furthermore, the sensor’s frequency response was tested across a frequency range of 50 Hz to 20 kHz, demonstrating that the measurement system can effectively respond within the sensor’s operational frequency range. Additionally, temperature measurement experiments showed a goodness of fit of 0.997 for the central wavelength of the FBG as the temperature increased. This research indicates that the improved optical path connection method not only accomplishes a synchronous demodulation of both temperature and voltage parameters but also markedly enhances the linearity and resolution of the voltage sensor. This discovery offers novel insights for further refining sensor performance and broadening the applications of optical voltage sensors.

## 1. Introduction

With the increasing demand for high-precision and high-reliability voltage measurements in power systems, traditional voltage measurement methods have gradually shown limitations in the face of complex environments and high voltage levels. In recent years, optical fiber sensing and optical communication network technologies have experienced rapid development, and different optical fiber sensing technologies have been successfully applied in various application fields. Optical fiber sensors provide a promising alternative to electronic sensors due to their distinct advantages of sensitivity, linearity, resistance to drift, inherent electric safety, and electromagnetic interference [1]. Therefore, new voltage sensors based on a fiber Bragg grating (FBG) and piezoelectric ceramics (PZTs) have emerged as an effective means to solve these problems. A PZT-FBG (fiber Bragg grating–piezoelectric ceramic) voltage sensor not only has the advantages of traditional sensors but also combines the technical advantages of the two, and has become a new type of sensor with broad application prospects. As a highly sensitive optical sensor, FBG can reflect the small changes in external physical quantities by monitoring the change in Bragg wavelength. PZT is a kind of functional material with a piezoelectric effect, which will undergo mechanical deformation under the action of applied voltage. In fact, based on low cost and easy processing, the piezoelectric effect and inverse piezoelectric effect of piezoelectric ceramics are widely used as electric field sensors or current sensors for power systems together with other effects [2,3,4,5,6,7]. The combination of the two can achieve an indirect measurement of the voltage. When the voltage is applied, the PZT element deforms, which in turn causes the strain of the FBG and ultimately leads to the drift of the Bragg wavelength. By accurately measuring the wavelength drift, the applied voltage value can be calculated. This design not only improves the accuracy and stability of the voltage measurement but also effectively overcomes the shortcomings of traditional sensors in electromagnetic interference.

At present, researchers attach great importance to voltage monitoring methods using optical elements or electro-optic materials. They can use optical fiber to achieve a robust long-distance data transmission of up to 30 km, providing broadband bandwidth and excellent electromagnetic interference immunity [8]; when integrated with the optical cable composite ground wire (OPGW), the sensor can efficiently measure the grid voltage [9]; and the measurement range can be extended to 132 kV by combining with a voltage divider or a larger size PZT element [10,11]. Liu et al. achieved a significant reduction in spectral shift variations by 50.86–87.82% through the implementation of a flexible coupling structure, enabling linear voltage sensing up to 6 kV [12]. Based on FBG-PZT, some researchers have proposed electro-optic voltage sensors. Yao Y et al. [13] proposed an FBG-PZT sensor for DC and AC voltage measurements, with a linear range of 0~500 V and a wavelength drift of 0~34 pm. Dante A et al. [14] introduced a temperature-independent FBG-PZT voltage sensor. Using a dual-grating scheme and a low-voltage monolithic multi-layer piezoelectric actuator (MMPA), an accurate measurement of AC signals over wide temperature ranges from −29 °C to +90 °C was achieved. Ribeiro B et al. [15] developed an optical voltage transformer (VT) based on FBG and PZT for a 13.8 kV distribution system. The experimental results show that there is a linear relationship between the applied voltage and the displacement of the FBG reflection spectrum. Pacheco M et al. [16] developed a high-pressure sensor using a traditional piezoelectric disk to drive a fiber Bragg grating that is mechanically fixed along the radial direction. Wu et al. [17] proposed and experimentally demonstrated a high-sensitivity voltage sensor based on an optoelectronic oscillator (OEO). An equivalent phase-shifted fiber Bragg grating (EPS-FBG) with a narrow notch in the reflection spectrum is introduced into the OEO. Using an electrical spectrum analyzer or a digital signal processor (DSP) to monitor the drift of the OEO oscillation frequency, a high-speed and high-resolution voltage measurement can be achieved. The experimental results show that the voltage sensitivity is 0.55 GHz/V. Gonçalves M N et al. [18] constructed an optical voltage transformer based on a fiber Bragg grating (FBG) and piezoelectric ceramic (PZT). The results show that the proposed OVT can reproduce up to 41 harmonics without obvious distortion and a pulse surge up to 2.5 kHz.

Generally speaking, in the PZT-FBG sensor configuration, the FBG (fiber Bragg grating) shows a high sensitivity to temperature parameters. Therefore, in the PZT-FBG sensing system, a series of measures are usually taken to compensate or eliminate the influence of temperature on the performance of FBG. Garção et al. [19] developed an FBG-based optical current transformer. By using a pair of FBGs for thermal compensation in the measurement system and using the second FBG as a fixed filter, the dual-grating demodulation technology is realized and the implementation cost is reduced. For OVT based on FBG-PZT, Yang et al. [20] proposed a bi-piezoelectric structure to eliminate the influence of temperature. The voltage is not applied to the reference structure, but only to the sensor structure, and both are affected by temperature changes. Some researchers continue to explore a non-traditional strategy that uses passive technology to compensate for temperature changes using only a single FBG (fiber Bragg grating) [21,22]. This method is called the biomaterial effect method. The basic principle is to incorporate specific materials with different thermal expansion coefficients (CTEs) into the sensor structure. Hsu et al. [23] proposed that the thermal expansion of the selected material must be exactly equivalent to the expansion of the FBG (fiber Bragg grating) induced by the thermal expansion coefficient of silica, so as to ensure that the generated negative strain (leading to the blue shift of Bragg wavelength) can effectively offset the inherent thermal red shift effect of the FBG. For an FBG-based optical voltage transformer (OVT), Fusiek et al. [24] proposed a scheme using an FBG with double reflection peaks and twice the length of the piezoelectric element. In this configuration, half of the FBG is fixed on the piezoelectric material, while the other half is combined with an extended electrode with a thermal expansion coefficient similar to the ceramic material. Cheng X et al. developed a calibration algorithm that addresses the effects of temperature on sensor sensitivity and guided wave propagation [25]. Gonçalves M N et al. [10] adopted a passive temperature compensation method based on the thermal expansion of the material, and designed a mechanical structure of an OVT to make the expansion of the material compensate the thermal expansion of the FBG. However, the above research based on a PZT-FBG voltage sensor only realizes the single parameter measurement of voltage. In the above research, the method of using the thermal expansion of the material to eliminate the influence of temperature needs to accurately calculate the thermal expansion coefficient of the material and the structural parameters of the sensor to ensure the effectiveness of temperature compensation, which increases the difficulty and complexity of the design. When the dual-grating demodulation method is used to eliminate the influence of temperature, the temperature change can be accurately measured by introducing a reference FBG that is only affected by temperature, and it can be separated from the measured strain signal, thereby improving the measurement accuracy. However, an additional FBG will be used for temperature compensation, and the FBG is not fully utilized.

Based on the above research, this paper proposes a PZT-FBG voltage–temperature demodulation optical path architecture based on the previous work and research of dual-grating spectral overlapping demodulation technology applied to temperature-independent piezoelectric grating voltage sensors. The scheme realizes the effective utilization of the idle temperature compensation reference grating in the double grating method, and converts it into a temperature measurement grating. The scheme proposed in this paper not only optimizes the resource utilization but also realizes the synchronous measurement of the two key parameters of voltage and temperature.

## 2. Principle and Design of Sensors

Previous work and research have shown that the relationship between the FBG center wavelength and the voltage in the temperature-independent piezoelectric grating voltage sensor can be described as Formula (1). The demodulation principle and sensing principle of the dual-grating spectral overlapping demodulation technology applied to the temperature-independent piezoelectric grating voltage sensor can be obtained by [12].

(1) $$\frac{\Delta \lambda_{C}}{\Delta U}=\left(1-\rho_{e}\right)\frac{nd_{33}\lambda_{C}}{L_{FBG}}$$

**Figure 1:**
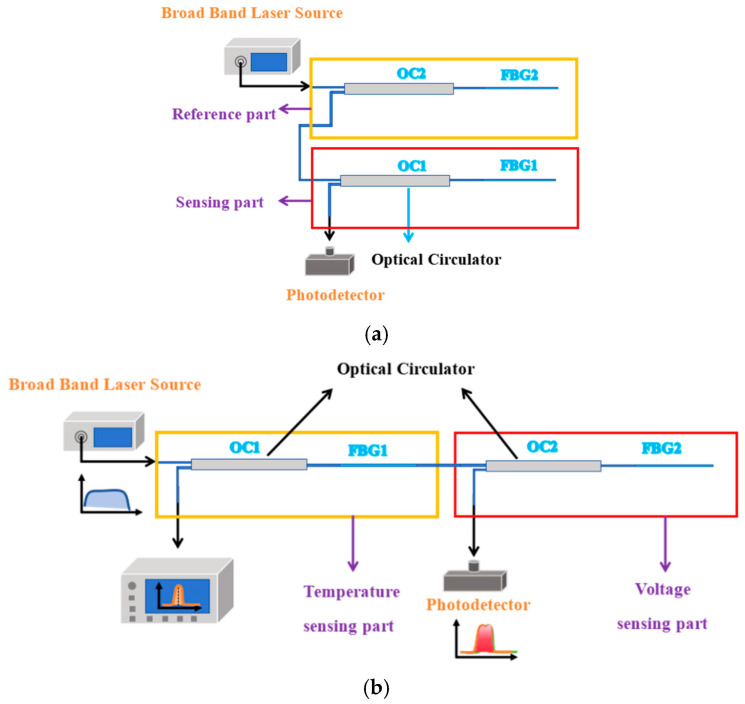
(**a**) The optical path connection before optimization; (**b**) optimized optical path connection.

**Figure 2:**
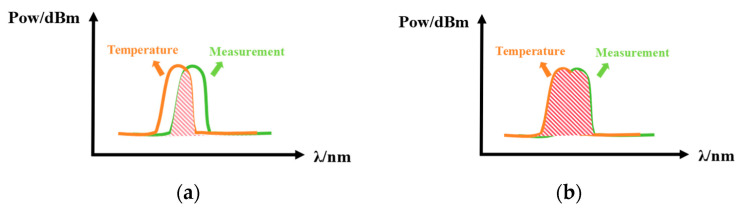
(**a**) The spectral overlap area before optimization; (**b**) optimized spectral overlap area.

In order to eliminate the influence of temperature, a dual piezoelectric sensor structure has been designed in previous studies, as shown in Figure 1a. The sensor can be divided into a reference part and a sensing part. The reference part includes an optical circulator 1 and a PZT-FBG1, in which the piezoelectric ceramic does not apply voltage, and is mainly set up to compensate for the temperature effect; the sensing part includes an optical circulator 2 and a PZT-FBG2, where the piezoelectric ceramic is applied to the voltage to be measured. The center wavelengths of the two FBGs in the reference part and the measurement part are similar. The spectrum of the light incident from the broadband laser source will change in the optical path. The light enters FBG1 after passing through the circulator 1, and the light reflected by FBG1 enters the optical circulator 2 and FBG2 in turn. Finally, the light reflected by FBG1 and FBG2 enters the optical detection. Since the two FBGs made of the same material are placed in the same environment, the thermal expansion coefficient α and the thermo-optic coefficient η are also the same, so when the temperature changes, the two FBGs will undergo the same deformation. The output light depends on the overlapping area of the two FBG spectra. The overlapping area is shown in Figure 2a, and its value is only related to the relative center wavelength position of the FBG, but not to its absolute center wavelength position.

(2) $$M_{PD}\left(\lambda \right)=0.95\times {\left(1-k\right)}^{2}\int_{aa}^{bb}I_{PD}d\lambda$$

(3) $$M_{PD}\left(\lambda \right)=0.95\times {\left(1-k\right)}^{2}\int_{aa}^{bb}S\left(\lambda_{B}\right)F_{R2}\left(\lambda \right)F_{S2}\left(\lambda \right)d\lambda .$$

The optical path connection of the optimized double-grating piezoelectric module is shown in Figure 1b. The sensor is still divided into two parts. One is the temperature measurement part, including the optical circulator 1 and PZT-FBG1, in which the ceramic is not voltage, and is mainly set up to measure the temperature. The other part is the voltage sensing part, including the optical circulator 2 and PZT-FBG2, in which the piezoelectric ceramic is applied to the voltage to be measured. The center wavelengths of the two FBGs in the reference part and the measurement part are similar.

As illustrated in Figure 1b, the spectrum of light emitted from the broadband laser source undergoes modifications as it propagates through the optical path. The light first passes through Circulator 1 and enters FBG1. The transmitted light from FBG1 subsequently propagates through Circulator 2 and FBG2. Ultimately, the light reflected by FBG1 and FBG2 is directed to the optical detection system, while the light reflected solely by FBG1 is routed to the spectrometer.

Given that both FBGs are fabricated from the same material and are subjected to identical environmental conditions, they share the same thermal expansion coefficient α and thermo-optic coefficient η. Consequently, any temperature variation induces identical deformations in both FBGs, thereby enabling the elimination of temperature effects during voltage measurements. Since FBG1 is solely influenced by temperature, the wavelength shift of its reflected spectrum corresponds directly to temperature changes, allowing for a precise temperature measurement. The light detected in the voltage sensing component depends on the overlapping region of the spectra from the two FBGs. As depicted in Figure 2b, the magnitude of this overlapping area is determined solely by the relative central wavelength positions of the two FBGs and is independent of their absolute central wavelength positions.

The spectrum provided by the broadband laser source can be approximated by the Gaussian equation.

(4) $$S\left(\lambda_{B}\right)=I_{peak}exp\left[-4\left(ln2\right){\left(\frac{\lambda -\lambda_{0}}{\Delta \lambda_{0}}\right)}^{2}\right]$$

The *I_peak_* is the peak power, the center wavelength, and the full width at half maximum (FWHM).

*I_peak_* is defined as follows:

(5) $$I_{peak}=\frac{P_{0}}{\Delta \lambda_{0}}{\left(4ln\frac{2}{\pi }\right)}^{\frac{1}{2}}$$

Considering the central wavelength of the FBG, the reflectivity of the FBG is simplified by the Gaussian equation. The reflectivity of the FBG in FBG1 and FBG2 can be expressed as Formulas (6) and (7).

(6) $$F_{FBG_{1}}\left(\lambda \right)=R_{0}exp\left[-4\left(ln2\right){\left(\frac{\lambda -\lambda_{FBG_{1}}}{\Delta \lambda_{FBG_{1}}}\right)}^{2}\right]$$

(7) $$F_{FBG_{2}}\left(\lambda \right)=R_{0}exp\left[-4\left(ln2\right){\left(\frac{\lambda -\lambda_{FBG_{2}}}{\Delta \lambda_{FBG_{2}}}\right)}^{2}\right]$$

In the formula, $F_{FBG_{1}}(\lambda )$ represents the reflection spectrum of the temperature measurement unit, and $F_{FBG_{2}}(\lambda )$ represents the reflection spectrum of the voltage measurement unit. $\lambda_{FBG_{1}}$ and $\lambda_{FBG_{2}}$ represent the central wavelength of the fiber grating, $\Delta \lambda_{FBG_{1}}$ and $\Delta \lambda_{FBG_{2}}$ represent the FWHM of the fiber grating spectrum, and $R_{0}$ represents the maximum reflectivity of the fiber grating.

Therefore, for the fiber Bragg grating in the temperature measurement unit, the reflected light $I_{1}(\lambda )$ should be the product of the incident broadband light source spectrum and the reflection spectrum of the reference grating, which can be described as Formula (8).

(8) $$I_{1}\left(\lambda \right)=S\left(\lambda_{B}\right)F_{R2}\left(\lambda \right)$$

The reflected light of FBG2 is the product of the transmitted light of FBG1 and the reflectivity of FBG2, which can be described as Formula (9).

(9) $$I_{2}\left(\lambda \right)=\left[\begin{array}{c} S\left(\lambda_{B}\right)-S\left(\lambda_{B}\right)F_{R1}\left(\lambda \right) \end{array}\right]F_{S2}\left(\begin{array}{c} \lambda \end{array}\right)$$

(10) $$I_{PD}=I_{2}\left(\lambda \right)$$

(11) $$I_{PD}=\left[\begin{array}{c} S\left(\lambda_{B}\right)-S\left(\lambda_{B}\right)F_{R2}\left(\lambda \right) \end{array}\right]F_{S2}\left(\begin{array}{c} \lambda \end{array}\right)$$

The signal received in the photodetector can be expressed in the form shown in Formula (11). It is worth noting that the signal received in the photodetector is closely related to the central wavelength of the FBG, especially FBG2. Any change in the central wavelength will be directly reflected in the change in the output light intensity. Taking into account the light intensity loss k that may occur when light enters the grating, and the photoresponsivity of the photodetector provided by the manufacturer near 1550 nm (wavelength range 1400 nm to 1650 nm) with a light responsivity of 0.95 A/W, the optical signal received by the photodetector can be further described by Equation (13).

(12) $$M_{PD}\left(\lambda \right)=0.95\times {\left(1-k\right)}^{2}\int_{aa}^{bb}I_{PD}d\lambda$$

(13) $$M_{PD}\left(\lambda \right)=0.95\times {\left(1-k\right)}^{2}\int_{aa}^{bb}\left(\left[S\left(\lambda_{B}\right)-S\left(\lambda_{B}\right)F_{FBG_{1}}\left(\lambda \right)\right]F_{FBG_{2}}\left(\lambda \right)\right)d\lambda$$

where $S(\lambda_{B})$ is the spectrum provided by the broadband laser source, and $F_{FBG_{1}}(\lambda )$ and $F_{FBG_{2}}\left(\lambda \right)$ are the reflectivity of the FBG in FBG1 and FBG2, respectively. The upper and lower bounds of the integral are determined by the light range provided by the broadband laser source. $\lambda_{FBG_{1}}$ and $\lambda_{FBG_{2}}$ denote their central wavelengths, $\Delta \lambda_{FBG_{1}}$ and $\Delta \lambda_{FBG_{2}}$ denote their full width at half maximum, respectively, and $R_{0}$ is their maximum refractive index.

Based on the above theoretical analysis, the intensity of the transmission spectrum used after the optimization of the optical path is much larger than the reflected light intensity before the optimization, and the overlap area entering the optical detection is also increased.

## 3. Experiments and Results

**Figure 3:**
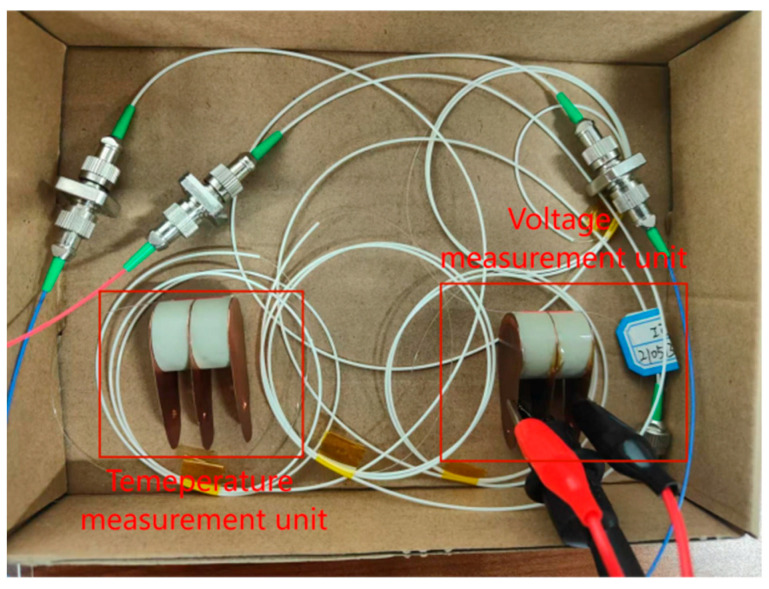
The optimized physical diagram of the PZT-FBG voltage/temperature sensor.

**Figure 4:**
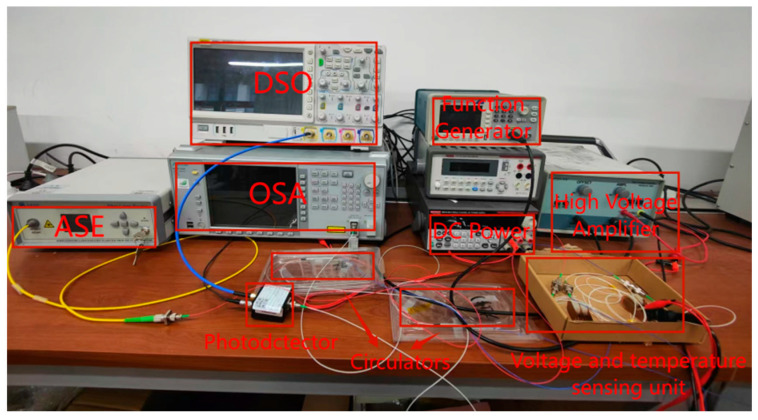
Experimental test platform.

Based on the aforementioned sensor principle, the optimized sensing system is illustrated in Figure 3. As shown in Figure 4, the test platform architecture is designed for the sensor system, integrating key components such as a signal generator, a high-voltage amplifier with a gain of 500, a broadband light source, and a Thorlabs PDA 10 CS-EC photodetector (Beijing Keyang Photonics Technology Co., Ltd., Beijing, China). The oscilloscope used in the experiments is model AFG1022, manufactured by Tektronix (Beaverton, OR, USA), and the optical spectrum analyzer is model MS9740A, manufactured by Anritsu (Atsugi, Kanagawa, Japan). The dimensions of the piezoelectric ceramic tube are as follows: outer diameter 20 mm, inner diameter 15 mm, and height 10 mm. The experiments were conducted at room temperature (23 ± 2 °C). In this system, the broadband laser source and the signal generator serve as the input signal sources. The voltage signal generated by the signal generator is amplified 500 times by the high-voltage amplifier before being fed into the sensor system. Subsequently, the output signal from the sensor system is directed to the photodetector for conversion and finally displayed on the oscilloscope. As can be seen from Figure 3, the design of the sensor and its verification system is not complex. More importantly, the sensing and reference parts adopt exactly the same structure and materials, thereby avoiding potential errors introduced by design differences. Additionally, the design of the sensor structure ingeniously eliminates the influence of temperature fluctuations on the measurement results, enhancing the stability and accuracy of the system.

### 3.1. Three Typical Voltage Signals’ Test Results

**Figure 5:**
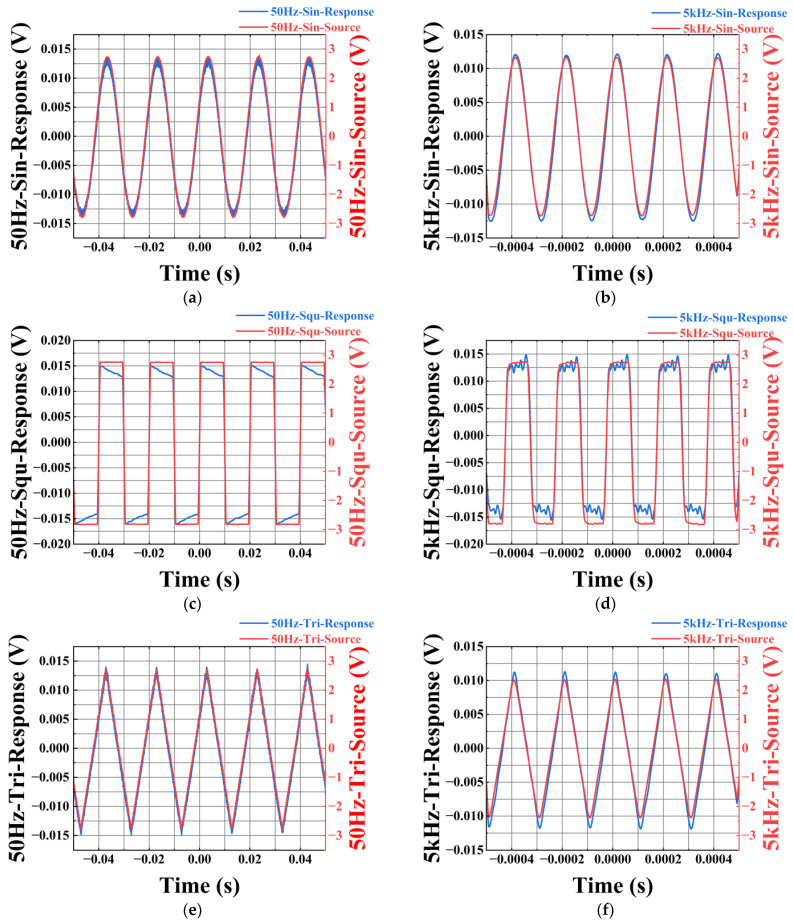
(**a**) 1.2 kV, 50 Hz sine wave input and output response; (**b**) 1.2 kV, 5 kHz sine wave input and output response; (**c**) 1.2 kV, 50 Hz rectangular wave input and output response; (**d**) 1.2 kV, 5 kHz rectangular wave input and output response; (**e**) 1.2 kV, 50 Hz triangular wave input and output response; (**f**) 1.2 kV, 5 kHz triangular wave input–output response.

The sine wave, square wave, and triangular wave are re-verified by using the optimized double grating piezoelectric module optical path connection method, and the results are shown in Figure 5. When testing the optimized sensing system, in order to compare the difference between the output characteristics of the sensor before and after optimization, the PZT, FBG, and experimental test instruments used in this comparison experiment are the same. The voltage signal used in the experiment is an AC voltage signal with an amplitude of 1.2 kV amplified by a high-voltage amplifier. Figure 5a,c,e illustrate the response characteristics of the optimized sensor to three different waveforms at a frequency of 50 Hz. As shown in the figures, the input signal (blue line) after amplification by the voltage amplifier and the signal detected by the sensor (red line) exhibit high consistency in both waveform and phase, indicating that the sensor can accurately capture the features of the input signal under low-frequency conditions. Figure 5b,d,f further demonstrate the sensor’s response characteristics at a high frequency of 5 kHz. Despite the significant increase in frequency, the input signal (blue line) and the sensor response (red line) still maintain strong consistency, suggesting that the sensor also possesses excellent signal detection capabilities under high-frequency conditions. This consistency is reflected not only in the waveform shape but also in the amplitude and phase relationship of the signals, further validating the sensor’s reliability and accuracy across a wide frequency range. In summary, the optimized sensor demonstrates highly consistent response characteristics with the input signal under both low-frequency (50 Hz) and high-frequency (5 kHz) conditions, proving its superior performance across a broad frequency range. The reason for the distortion of the signal detected by the sensor is that the piezoelectric ceramic has a lag effect, and the square wave distortion is more obvious because when the piezoelectric ceramic encounters the turning point of the square wave, the signal changes drastically in a short time, which requires the piezoelectric ceramic to respond quickly, but in fact, the piezoelectric ceramic may not respond immediately.

**Figure 6:**
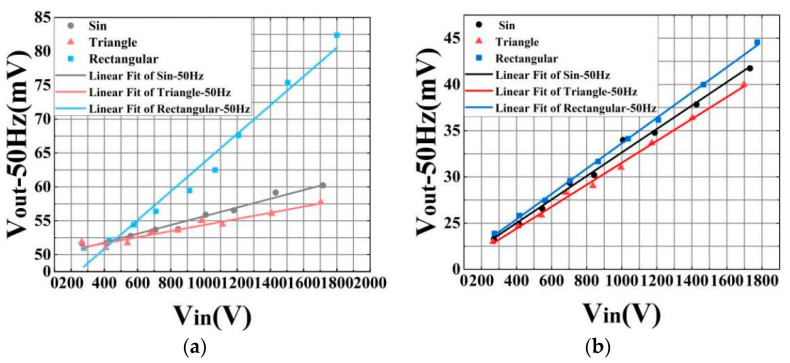
(**a**) Output fitting results of three basic waveforms at 50 Hz before optimization; (**b**) the output fitting results of three basic waveforms at 50 Hz after optimization.

The linearity test results of the sensor at 50 Hz under three different waveforms, both before and after the optimization of the optical path connection, are presented in Figure 6. Figure 6a illustrates the linear fitting results of the sensor under the three waveforms at 50 Hz prior to optimization. In this figure, the black dots denote the data captured by the sensor under a sine wave, the red triangles represent the data under a triangular wave, and the blue rectangles indicate the data under a rectangular wave. The corresponding fitted lines for these data points are shown in black, red, and blue, respectively. The R^2^ values for these lines are 0.975, 0.938, and 0.973, respectively. Post-optimization, the linear test results of the sensor under the same three waveforms at 50 Hz are depicted in Figure 6b. Here, the black dots again represent the sine wave data, the red triangles the triangular wave data, and the blue rectangles the rectangular wave data. The fitted lines for these data sets are also shown in black, red, and blue, with R^2^ values of 0.996, 0.999, and 0.992, respectively.

The experimental results clearly demonstrate a significant improvement in the output linearity of the sensor following optimization, with the most notable enhancement observed under the triangular wave. Additionally, Figure 6 reveals that the optimized sensor enhances the resolution of voltage sensing under the same conditions, particularly under sine and triangular waveforms. This improvement is attributed to the significantly higher light intensity of the transmission spectrum compared to the reflection spectrum, which effectively reduces the impact of noise on the experimental outcomes.

Due to the limitations of the voltage amplifier available in the laboratory, the voltage measurement range in this study was constrained to 250–1800 V. However, in our previous work, we successfully achieved a 5 kV voltage measurement using a 500-fold voltage amplifier. Furthermore, we proposed a variable-stiffness flexible structure that extended the voltage measurement range of the PZT-FBG voltage sensor to 0–6 kV. By employing this flexible coupling structure for linear voltage sensing, the spectral shift was reduced by 50.86–87.82%.

### 3.2. Frequency Response Test of the Sensor and Temperature Measurements

**Figure 7:**
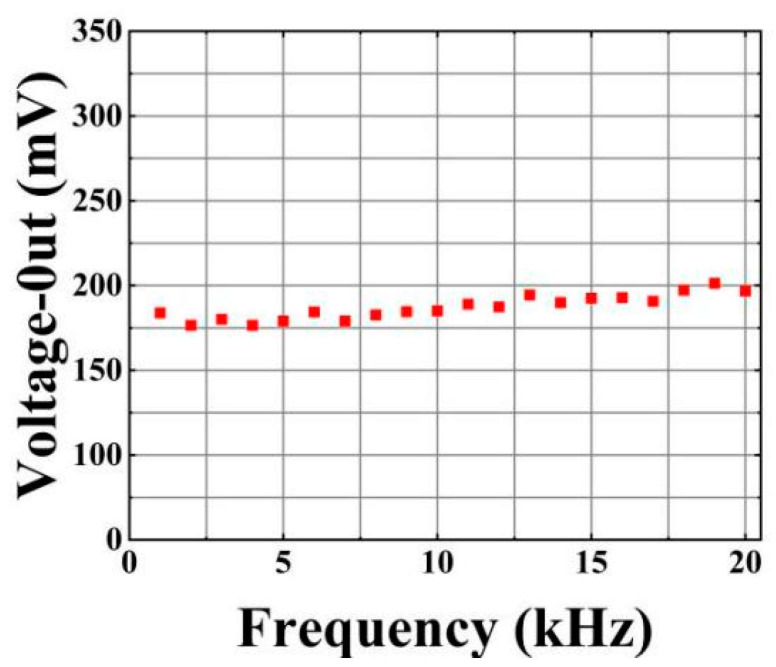
Frequency response test results.

Figure 7 shows the frequency response test results of the sensor with optimized optical path connection in the frequency range of 50 Hz to 20 kHz. This test is carried out on a unified test platform, and the voltage applied during the test is 1 kV. Overall, the sensor exhibits a fairly stable frequency response characteristic, which ensures its effective application in the measurement frequency range of 50 Hz to 20 kHz.

**Figure 8:**
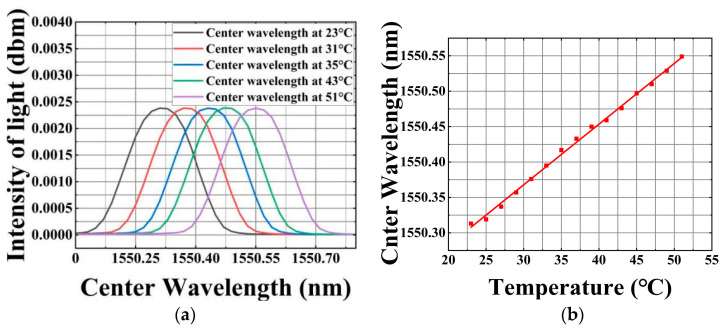
(**a**) The central wavelength shifts to the right with the increase in temperature. (**b**) Fitting results of center wavelength with increasing temperature.

The experimental setup of the temperature measurement is shown in Figure 8. In order to ensure a more accurate temperature environment, the sensor system is placed in a box and placed in a drying oven as a whole. The experimental temperature range was set to 23 °C to 51 °C, and the temperature difference between adjacent test points was set to 2 °C. At different temperatures (23 °C, 31 °C, 35 °C, 43 °C, and 51 °C), the central wavelength of the FBG (fiber Bragg grating) will shift, which is shown in Figure 8a. Specifically, the center wavelength of the FBG moves to the long wavelength with the increase in temperature. After fitting these central wavelength scatter data offset by the temperature rise, the results are shown in Figure 8b. The goodness of fit is as high as 0.997, which indicates that there is an excellent linear relationship between temperature and FBG center wavelength. Therefore, the temperature can be measured by measuring the offset of the FBG center wavelength.

In addition to the performance demonstrated within the tested temperature range (23 °C to 51 °C), it is crucial to consider the potential calibration challenges and long-term stability of the sensor under extreme environmental conditions. For instance, in applications where the sensor is exposed to temperatures significantly beyond the tested range or to rapid thermal cycling, the linear relationship between temperature and the FBG center wavelength may deviate due to limitations in material thermal expansion or the degradation of the FBG and PZT materials. Furthermore, prolonged exposure to high temperatures or harsh environmental conditions, such as high humidity, corrosive atmospheres, or mechanical stress, could lead to signal drift or material aging, potentially compromising the sensor’s accuracy and reliability. To address these challenges, future work will focus on evaluating the sensor’s performance under extreme conditions, including extended temperature ranges, thermal shock tests, and long-term stability assessments. Additionally, we plan to explore the selection of different materials and the continuous optimization of the sensor structure to enhance its adaptability to various environments, thereby improving the sensor’s stability and robustness. These efforts will be essential for ensuring reliable performance in demanding applications, such as power grid monitoring or aerospace systems, where extreme conditions are frequently encountered.

## 4. Discussion

In this paper, an optimized optical path connection method for a PZT-FBG (piezoelectric ceramic–fiber Bragg grating) voltage sensor is proposed. This connection method not only fully utilizes the original reference grating but also significantly enhances the performance of the sensor. The key optimization strategy lies in successfully converting the reference grating in the original dual-grating demodulation method into a temperature measurement grating. Experimental data show that the goodness of fit for the central wavelength scatter data reaches as high as 0.997 at different temperatures, thereby achieving a simultaneous measurement of temperature and voltage parameters. Notably, the intensity of the transmission spectrum used after the optical path optimization is significantly greater than the reflected light intensity before optimization, and the overlapping area of the light probe is also increased. The experimental results demonstrate that this optimization strategy not only significantly improves the output linearity and resolution of the PZT-FBG voltage sensor but also enhances sensor performance while maintaining structural simplicity without introducing additional mechanical components.

This study provides significant theoretical insights and experimental validation for the optimization of piezoelectric fiber Bragg grating voltage sensors, laying a solid foundation for further application research and development. However, due to limitations in experimental conditions and time, we have not yet conducted comprehensive performance testing in practical environments.

In future research, we aim to eliminate the reliance on temperature demodulation instruments. By integrating dual-grating signal processing technology with photodetectors, a direct temperature measurement can be achieved, thereby removing the need for demodulation instruments. Additionally, we plan to systematically investigate the long-term stability, signal drift characteristics, and the impact of material aging on the sensor’s performance, with the goal of comprehensively evaluating its reliability and durability in complex environments. These studies will provide more robust theoretical support and experimental data for the practical engineering applications of the sensor.

## 5. Conclusions

The experimental data demonstrate that the goodness of fit for the central wavelength scatter data reaches up to 0.997 at different temperatures, enabling accurate simultaneous measurements of both temperature and voltage parameters. Additionally, the optimized optical path shows a substantially higher transmission spectrum intensity compared to the reflected light intensity before optimization, with an increased overlap area of the light probe. These improvements result in the enhanced output linearity and resolution of the PZT-FBG voltage sensor while maintaining structural simplicity and avoiding the introduction of additional mechanical components.

## Data Availability

The original contributions presented in this study are included in the article. Further inquiries can be directed to the corresponding author.
